# Peer Review and Publication of Research Protocols and Proposals: A Role for Open Access Journals

**DOI:** 10.2196/jmir.6.3.e37

**Published:** 2004-09-30

**Authors:** Gunther Eysenbach

**Affiliations:** ^1^Centre for Global eHealth InnovationTorontoCanada

**Keywords:** Access to information, information dissemination, Internet, publishing, research design

## Abstract

Peer-review and publication of research protocols offer several advantages to all parties involved. Among these are the following opportunities for authors: external expert opinion on the methods, demonstration to funding agencies of prior expert review of the protocol, proof of priority of ideas and methods, and solicitation of potential collaborators. We think that review and publication of protocols is an important role for Open Access journals. Because of their electronic form, openness for readers, and author-pays business model, they are better suited than traditional journals to ensure the sustainability and quality of protocol reviews and publications. In this editorial, we describe the workflow for investigators in eHealth research, from protocol submission to a funding agency, to protocol review and (optionally) publication at JMIR, to registration of trials at the International eHealth Study Registry (IESR), and to publication of the report. One innovation at JMIR is that protocol peer reviewers will be paid a honorarium, which will be drawn partly from a new submission fee for protocol reviews. Separating the article processing fee into a submission and a publishing fee will allow authors to opt for “peer-review only” (without subsequent publication) at reduced costs, if they wish to await a funding decision or for other reasons decide not to make the protocol public.

It has long been advocated that journals take on a more active role in the “primary prevention” of poor research not only by peer reviewing final reports but by becoming involved earlier in the process through reviewing research protocols [1-3]. Even though some protocols are reviewed at a funding agency, it is also a fact that many projects (in particular smaller projects in eHealth) are never subjected to this scrutiny. Even if projects receive funding agency assessment, researchers and society may still benefit from a prior peer review and possible subsequent Medline-indexed publication. The arguments for doing so include the following [1]:

- highlighting good-quality studies at an early stage
- contribution to a register of selected trials, to reduce publication bias against negative (neutral) or inconvenient findings
- promotion of recruitment of cooperating centres and trial participants
- helping researchers in funding applications
- prevention of poor research
- prevention of data dredging by documentation of intended analyses
- establishment of priority of an important idea

In addition, the current development of journal editors asking for trial registration prior to enrollment of participants [4] places a renewed emphasis on the quality of the research protocol. Research protocols will undergo more scrutiny in the future [5] as peer reviewers of trial reports, having access to some key points from the protocol through the trial registry entry, will be able to read the submitted paper in the context of what had been proposed originally.

JMIR now also encourages submissions of protocols for peer review and (optionally) subsequent publication. This, in conjunction with its newly established study register, will increase the possibility of other researchers (such as systematic reviewers) finding negative and ongoing studies. It is also part of a larger vision of making JMIR a one-stop-shopping site by offering services for eHealth researchers at all stages of the knowledge production and dissemination cycle.

**Figure 1:**
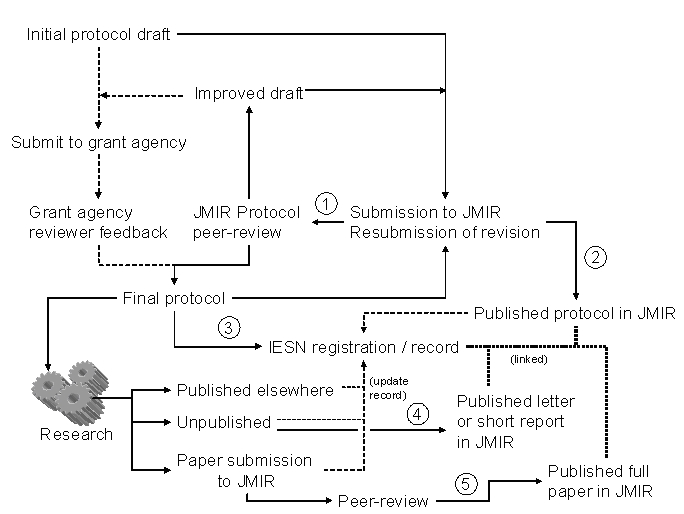
Possible “workflow” from the conception of an eHealth study to its publication

The possible “workflow” from study conception to publication is shown in the figure. Authors have the opportunity of submitting a research protocol for peer review to JMIR (point 1 in figure) either before or after submitting it to the funding agency. Peer review at JMIR will encompass suggestions for improvement and an expert opinion on the value of the research plan. Authors may either incorporate the suggested changes and resubmit the revised version, or publish the unchanged protocol alongside the peer-review report (2), or opt to refrain from publication. In addition, authors are, under the new policy of most medical journals, now required to register their studies. This can be done at the new International eHealth Study Register (IESR) located at JMIR [4]. The registry will assign a unique IESN (International eHealth Study Number) to the study and create a database entry summarizing some of the study information, including links to the published protocol or subsequent publications. After study completion, authors may submit a full paper to JMIR (5) or other journals, or – if time-constraints prevent authors from writing a full paper – at least publish the database entry with a short comment on the results as a letter or short report in JMIR [4].

Peer reviewers of protocols will be asked to use different standards from those used for peer-reviewed articles. There will be no “accept” or “decline” decision except in cases where the protocol is off-topic (see journal scope) or is clearly ethically or scientifically flawed. Reviewers are asked to comment on the existence of potential flaws which might threaten the validity of the research, to make suggestions for overcoming these flaws if they exist, or to suggest minor improvements to the research plan or the writing. 

The peer review and optional publication of protocols will be separate processes, in that the author may have the protocol peer reviewed only and not proceed to publication. The option of “peer review only” might be used by some investigators to obtain peer-review input before submission to a funding body while reserving disclosure of the research plan until after funding. Others might prefer to publish in order to be able to cite a fully peer-reviewed research protocol in a funding proposal, while some might wish to use the publication option after success of the funding applications in order to claim priority of the research ideas outlined in the protocol.

Peer review and publication of protocols will have a different cost structure from regular article submissions to JMIR. For normal research papers, authors' institutions or authors pay an article processing fee (currently set at $750, payable at step 5 in the figure) only if the article is accepted for publication. The fee covers costs incurred both at peer-review and at publication. For protocol submissions only, JMIR is introducing a separated fee. A $250 levy, payable upon submission, will cover the costs of honoraria to peer reviewers, and a separate $500 fee will cover the copyediting and typesetting costs of the optional publication. JMIR needs to recover the costs of peer reviews of protocols which are not published, while researchers might view the submission fee as payment for value received in the peer review.

To our knowledge, JMIR is the first Open Access journal taking this critical step of levying a submission fee. To encourage the sustainability and quality of Open Access journals, the Science and Technology Committee of the House of Commons of the United Kingdom has in fact recommended this step. In its Report the Committee stresses, “The introduction of a submission fee would be an important step towards ensuring the quality of scientific publications and we strongly recommend that author-pays publishers introduce this system [paragraph 174 and recommendation 67] [6].”

As peer reviewers may find review of protocols less appealing than review of finished research, we will offer an honorarium as a small incentive. This will help maintain the quality of reviews and promote a quick turnaround time. Authors will have the opportunity to nominate specific reviewers whom we will approach first, but we reserve the right to replace them if they decline or seem unsuitable.

This model is an experiment, but we think it is viable. Protocol review and publication may become an important role for Open Access journals. Clear advantages flow to all parties in the process. Authors obtain external expert opinions on their methods and are able to show funding agencies reviewed protocols. They are also able to document priority of ideas and methods and to solicit potential collaborators. Open Access journals because of their electronic form, openness for readers and author-pays business model are better suited than traditional journals to provide the sustainability and quality of protocol review and publication.
